# A Simple Quantitative Bedside Test to Determine Methemoglobin

**DOI:** 10.1016/j.annemergmed.2009.07.022

**Published:** 2010-02

**Authors:** Fathima Shihana, Dhammika Menike Dissanayake, Nicholas Allan Buckley, Andrew Hamilton Dawson

**Affiliations:** a South Asian Clinical Toxicology Research Collaboration, Faculty of Medicine, University of Peradeniya, Peradeniya, Sri Lanka; b Department of Pathology, Faculty of Medicine, University of Peradeniya, Peradeniya, Sri Lanka; c The POW Clinical School, University of New South Wales, Sydney, Australia; d School of Population Health, University of Newcastle, Newcastle, Australia

## Abstract

**Study objective:**

Methemoglobinemia after pesticide poisoning is associated with a mortality of 12% in Sri Lanka. Treatment is complicated by the lack of laboratory facilities. We aimed to develop and validate a low-cost bedside test for quantitative estimation of clinically significant methemoglobin to be used in settings of limited resources.

**Methods:**

A method to reliably produce blood samples with 10% to 100% methemoglobin was developed. Freshly prepared methemoglobin samples were used to develop the color chart. One drop (10 μL) of prepared methemoglobin sample was placed on white absorbent paper and scanned using a flatbed Cannon Scan LiDE 25 scanner. The mean red, green, and blue values were measured with ImageJ 1.37v. These color values were used to prepare a color chart to be used at the bedside. Interobserver agreement was assessed against prepared samples. The results from clinical use were compared with formal methemoglobin measurements.

**Results:**

The red color value was linearly related to percentage methemoglobin (*R*^2^=0.9938), with no effect of absolute hemoglobin concentration. Mean interobserver (N=21) agreement and weighted κ for scanned methemoglobin spots using the color chart were 94% and 0.83, respectively. Mean interobserver (N=9) agreement and weighted κ for a freshly prepared methemoglobin sample with the chart were 88% and 0.71, respectively. Clinical use of the color chart also showed good agreement with spectrometric measurements.

**Conclusion:**

A color chart can be used to give a clinically useful quantitative estimate of methemoglobinemia.

## Introduction

Methemoglobinemia can be a life-threatening complication after exposure to a range of chemicals such as nitrates, nitrites, and aniline and drugs such as dapsone.^1-4^ Antidote treatment with methylene blue is generally effective and is enhanced by monitoring the response to treatment.^5^ In rural Asia, monitoring is unavailable and contributes to the high death rates after poisoning by some methemoglobinemia-inducing pesticides, in particular the herbicide propanil, which has a 12% mortality rate.^6,7^

The recommended dose of methylene blue for the treatment of methemoglobinemia is 1 to 2 mg/kg body weight, but recurrence is common and repeated dosing is required for some patients.^8^ However, doses of greater than 4 mg/kg methylene blue may actually exacerbate the methemoglobinemia, especially in patients with severe glucose-6-phosphate dehydrogenase deficiency.^9^ Because glucose-6-phosphate dehydrogenase deficiency affects between 3% and 4% of the Sri Lankan population, therapeutic uncertainty occurs when the clinical response is partial or unclear. This uncertainty may contribute to the high mortality.

Ideally, the treatment of methemoglobinemia should be guided by measurements of methemoglobin levels.^9^ The objective of this research was to develop and validate a low-cost bedside test for quantitative estimation of clinically significant methemoglobin to be used in settings of limited resources.

## Materials and Methods

A series of experiments was designed to assess the concentration of sodium nitrite necessary for the conversion of hemoglobin into methemoglobin in the range of 10% to 100%. Variable concentrations of methemoglobin were induced in normal blood samples by adding various concentrations of sodium nitrite prepared by diluting a 12.41 g/L sodium nitrite stock solution. An equal amount of diluted sodium nitrite was added to test tubes containing 1.0 mL of normal blood. The blood sample's methemoglobin concentration was measured using the method below, which is based on that of Evelyn and Malloy.^10^ Diluted methemoglobin blood samples were prepared by adding 0.2 mL methemoglobin blood into 10.0 mL of diluents containing phosphate buffer and detergent. The absorbance at 630 nm was used to determine the methemoglobin concentration.

Red–dark brown blood was observed in the 10% to 100% methemoglobin blood samples. Freshly prepared series of methemoglobin samples were placed on a piece of white absorbent material and scanned within 1 minute using a Canon Scan LiDE 25 color image scanner (Canon Inc, Vietnam). The color value (from red to brown) of scanned samples was measured using an open source image processing and analysis program ImageJ 1.37v, which allows the mean red, green, and blue values to be measured directly and then plotted against the measured methemoglobin levels.^11^ The mean red, green, and blue values were used to prepare a color chart that was subsequently printed.

The prepared color chart was validated using both scanned and freshly prepared methemoglobin blood samples. Physicians, preinternship medical graduates, and nonmedical office staff were used as these observers (n=21). Nine scanned methemoglobin spots (10% to 100%) were randomly given to observers, who were asked to estimate the methemoglobin percentage by matching the sample with a color chart labeled in 5% increments. The same methodology was used with 9 freshly prepared methemoglobin samples spotted on absorbent material. These observers (n=9) had to make an estimate of the methemoglobin percentage within 2 minutes. The testing was performed under fluorescent or tungsten lights, with a distance of about 30 cm from eye levels to color chart. The results were compared with the actual values of methemoglobin for both scanned and freshly prepared methemoglobin blood samples, and agreements of observers' results with the color chart were calculated with κ statistics.

The effect of a range of hemoglobin concentrations and methylene blue concentrations in the blood samples was also assessed in further validations of the color chart. Blood samples with an initial known methemoglobin concentration were incubated with methylene blue. Red, green, and blue values of the samples were measured: before methylene blue, just after addition of methylene blue, and after 1-hour incubation.

For clinical samples, clinicians used the chart and recorded the estimated methemoglobin percentage by using the color chart. Thirteen patients' methemoglobin levels were estimated with the color chart, and a blood sample taken at the same time was analyzed independently with spectrophotometer (Unico UV-Vis; model UV 2800).

The interrater agreement was estimated with the weighted κ statistic with Stata IC 10 (StataCorp, College Station, TX). The other analyses were conducted with GraphPad Prism 5, and graphs were made with the same software.

Ethics approval was obtained from the Ethical Review Committee of the University of Peradeniya, Sri Lanka.

## Results

**Figure 1:**
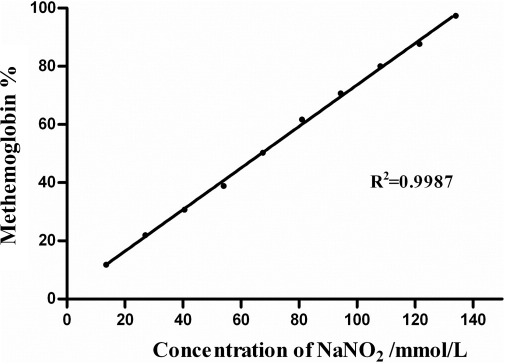
Amount of methemoglobin formed with increasing sodium nitrite concentration.

We created a set of blood samples with increasing (10% to 100%) methemoglobin concentration that showed a gradual increase in absorbance at a wavelength of 630 nm. The conversion of hemoglobin to methemoglobin increased linearly, with a corresponding increase of sodium nitrite concentration. The calibration plot (Figure 1) of methemoglobin versus sodium nitrite concentration yielded an *R*^2^ of 0.9987.

**Figure 2:**
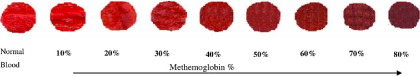
Samples used to measure red, green, and blue values of methemoglobin. A drop (10 μL) of blood was placed on white absorbent material and scanned with a flatbed Canon Scan LiDE 25 scanner.

**Figure 3:**
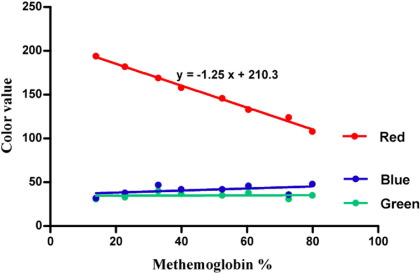
The variation of red, green, and blue color values with methemoglobin concentration (%) Scanned methemoglobin spots were analyzed with an Image J program to measure the mean color values.

An obvious change in color from red to dark brown was observed (Figure 2). Measuring the color value of the scanned blood drops showed an inverse relationship between red color value and methemoglobin content, with a correlation coefficient of 0.9938 (Figure 3). The red color value changed from 196 to 108 as the methemoglobin levels changed from 10% to 80%; the change in the green and blue color values was negligible.

**Figure 4:**
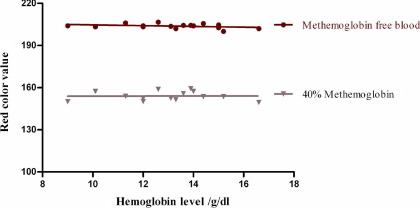
The change in the color value of normal and 40% methemoglobin blood samples. The red color value of blood samples with hemoglobin levels from 9 g/dL to 17 g/dL was measured in an Image J program.

Checking the effect of variation in hemoglobin concentration, we found a minimal effect on the red color value of both methemoglobin and methemoglobin-free blood samples in the range of 9 g/dL to 17 g/dL hemoglobin (Figure 4).

**Figure 5:**
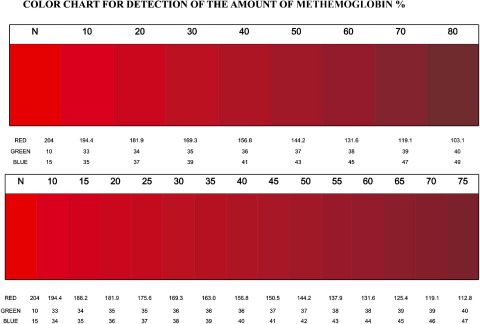
Color chart for the measurement of methemoglobin. The color chart was prepared according to the change in red, green, and blue value of blood samples with known methemoglobin concentration.

The color chart prepared from the RBG values of the standard methemoglobin blood samples is shown in Figure 5. The methemoglobin percentage represented by 9 scanned blood samples (blots) was estimated by 21 independent observers using the color chart. These test-scanned samples were based on spiked samples with a known methemoglobin concentration between 10% and 80%. There was excellent interobserver agreement for scanned methemoglobin samples with this color chart; mean interobserver agreement was 94%, and unweighted and weighted κ values were 0.37 and 0.83, respectively. The type of light (artificial versus natural) and time of day did not affect the observers' ability to estimate methemoglobin color. The same procedure with freshly prepared methemoglobin blood samples with 9 independent observers also showed high interobserver agreement (88%) and weighted κ (0.71).

**Figure 6:**
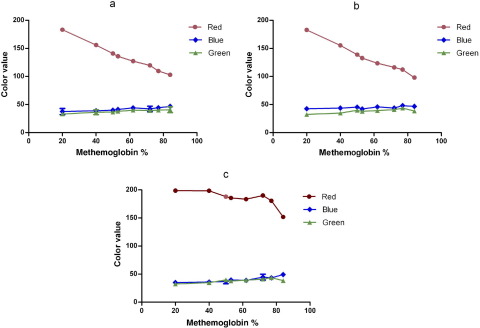
Demonstration of the change in red, green, and blue value of blood samples with methylene blue, starting from a known concentration of methemoglobin *A*, before methylene blue addition, *B*, just after methylene blue was added, *C*, after incubation with methylene blue for 1 hour.

The effect of administration of methylene blue on the color of the blood sample was also studied for further validation of the color chart. Blood samples with an initial known methemoglobin concentration were incubated with methylene blue. Red, green, and blue of the samples were measured before addition of methylene blue, at methylene blue addition, and after 1 hour of incubation (Figure 6). Although there was a significant change in blue color value at methylene blue addition, there was extremely low variation in blue values after an hour of incubation with methylene blue. The low variation at 1 hour corresponds to the time when most clinical samples are collected after administration of methylene blue to a patient.

**Figure 7:**
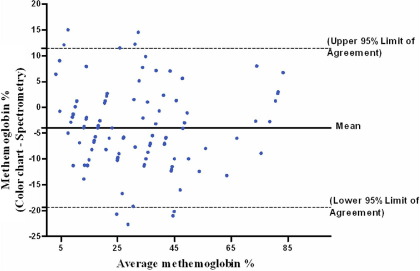
Bland-Altman plots of methemoglobin concentration estimated by spectrophotometric and color chart methods. The difference is plotted against the average of the two methods. Differences from the gold standard in future patient samples should lie between the limits 95% of the time.

The color chart was introduced to 3 hospitals, where 108 samples of methemoglobin concentrations were measured in 13 patients. Figure 7 shows the Bland-Altman plot for comparison of methemoglobin concentration measured by spectrophotometer and the color chart for these clinical samples.

## Discussion

We have developed and validated a simple bedside test that can be used to accurately estimate the concentration of methemoglobin in blood samples and guide clinical management. It should be useful in hospitals with limited resources or in situations in which methemoglobin concentrations cannot be measured rapidly.

Blood samples with a methemoglobin concentration greater than 20% have a noticeably brown coloration. Previous studies have shown that observers can detect the presence of methemoglobin by using a bedside blood spot test. However, no work has previously tested whether observers can quantify the methemoglobin concentration.^12^ Our results indicate that accurate estimates are possible.

The interobserver agreement of scanned methemoglobin blood samples with the color chart was very good. The unweighted κ suggests only moderate agreement in terms of the precise estimate.^13^ However, the weighted κ (which weighs the extent of disagreement) indicates extremely high agreement and that any difference in estimates was usually very minor. This is clinically important because it allows multiple observers to detect significant changes in a patient's methemoglobin concentration and thus allows treatment to be titrated against response. In clinical use, the color chart shows a good agreement in Bland-Altman plot in spite of the aggregation of methemoglobin concentrations in 5% (Figure 7). Because the agreement between the color chart and the spectrophotometric method in actual clinical use was high, the color chart can be used clinically for the same purposes as a formal laboratory measurement. Changes of more than 10% from a previous reading are likely to represent a true change in methemoglobin concentration (Figure 7).

Our results show that variability in hemoglobin levels or ambient light was not associated with significant variation in the methemoglobin color value. It seemed that any change in color perception affected both the chart and patient sample equally. Because scanners use a consistent light source, we can also assume that red, green, and blue values from scanned spots will always be consistent for a given scanner. However, the color value of the chart also depends on the printer inks and paper and might fade with time. We recommend that printed versions of the color chart periodically be rescanned with a scanner and checked against the original red, green, and blue values shown in Figures 3 and 5.

Although there is a potential effect of methylene blue on the color of the blood sample, we did not observe any significant effect 1 hour after administration. Even within an hour it is likely that a relative change in methemoglobin with methylene blue would still be detectable even if the actual estimates were less accurate. In any case, there is little clinical justification for taking repeated methemoglobin estimation within an hour of methylene blue administration.

Abnormal hemoglobins such as hemoglobin M may affect the color value because they are known to interfere with estimates by co-oximetry.^14^ Similarly, some causes of methemoglobin cause the formation of small amounts of sulfhemoglobin or carboxyhemoglobin. Further studies are required to resolve these issues, although they will affect relatively few patients.

The results of the study suggest that the red, green, and blue value of scanned blood samples accurately estimates the percentage of methemoglobin present. A color chart produced from these red, green, and blue values can be used to give a clinically meaningful quantitative estimate of the percentage of methemoglobin present in a blood sample taken from a poisoned patient.

## Editor's Capsule Summary

*What is already known on this topic*

The methemoglobin concentration is used to diagnose and guide treatment of methemoglobinemia, but in many countries instruments needed to measure methemoglobin are not readily available.

*What question this study addressed*

Can a color chart allow visual clinical estimation of the methemoglobin concentration at the bedside?

*What this study adds to our knowledge*

Interobserver agreement using the chart was 94% on blood samples with methemoglobin concentrations of 10% to 80%. Clinical testing in 3 hospitals also showed good agreement.

*How this might change clinical practice*

This color chart can facilitate more rapid diagnosis of methemoglobinemia and is of particular value in settings that lack analytic instruments capable of performing this task.

## References

[1] Singh H., Purnell E., Smith C. (2007). Mechanistic study on aniline-induced erythrocyte toxicity. Arh Hig Rada Toksikol.

[2] Anderson C.M., Woodside K.J., Spencer T.A. (2004). Methemoglobinemia: an unusual cause of postoperative cyanosis. J Vasc Surg.

[3] Camp N.E. (2007). Methemoglobinemia. J Emerg Nurs.

[4] Fernando R. (2007). Managment of Poisoning.

[5] Cattaneo A., Furiosi D., Bolis C. (1987). Acute poisoning caused by methemoglobinemia-inducing substances: 6 cases. Minerva Med.

[6] Eddleston M., Rajapakshe M., Roberts D. (2002). Severe propanil [N-(3,4-dichlorophenyl) propanamide] pesticide self-poisoning. J Toxicol Clin Toxicol.

[7] De Silva W.A., Bodinayake C.K. (1997). Propanil poisoning. Ceylon Med J.

[8] Roberts D.M., Heilmair R., Buckley N.A. (2009). Clinical outcomes and kinetics of propanil following acute self-poisoning: a prospective case series. BMC Clin Pharmacol.

[9] Watcha M.F., Connor M.T., Hing A.V. (1989). Pulse oximetry in methemoglobinemia. Am J Dis Child.

[10] Dacie J. (1984). Practical Haematology.

[11] Rasband W. ImageJ. v. 1.37. National Institutes of Health. http://rsbweb.nih.gov/ij/index.html.2006.

[12] Harvey J.W. (2006). Pathogenesis, laboratory diagnosis, and clinical implications of erythrocyte enzyme deficiencies in dogs, cats, and horses. Vet Clin Pathol.

[13] Chmura Kraemer H., Periyakoil V.S., Noda A. (2002). Kappa coefficients in medical research. Stat Med.

[14] Haymond S., Cariappa R., Eby C.S. (2005). Laboratory assessment of oxygenation in methemoglobinemia. Clin Chem.

